# Adapting the Interpersonal Quality in Family Planning care scale to assess patient perspectives on abortion care

**DOI:** 10.1186/s41687-018-0089-7

**Published:** 2019-01-21

**Authors:** Kyla Z. Donnelly, Christine Dehlendorf, Reiley Reed, Daniela Agusti, Rachel Thompson

**Affiliations:** 1 The Dartmouth Institute for Health Policy and Clinical Practice, Dartmouth College, 1 Medical Drive, Lebanon, NH 03756 USA; 2 UCSF Department of Family and Community Medicine, 1001 Potrero Avenue, San Francisco, CA 94110 USA; 3 UCSF Department of Epidemiology & Biostatistics, San Francisco, 94158 CA USA; 4 UCSF Department of Obstetrics, Gynecology, & Reproductive Sciences, San Francisco, 94158 CA USA; 5 Sydney School of Public Health, Faculty of Medicine and Health, The University of Sydney, Sydney, 2006 NSW Australia

**Keywords:** Abortion, Interpersonal care, Measurement, Patient-provider communication, Scale development

## Abstract

**Background:**

Women value receiving quality interpersonal care during abortion services, yet no measure exists to assess this outcome from patients’ perspectives. We sought to adapt the Interpersonal Quality in Family Planning care scale (Dehlendorf et al., American Journal of Obstetrics Gynaecology 10.1016/j.ajog.2016.01.173, 2016) for use in abortion care.

**Methods:**

We adapted items from the original scale for the abortion context, and conducted cognitive interviews to explore the acceptability, understandability, and importance of the adapted items. Adults who spoke English and/or Spanish, had an abortion in the past year, and lived in the US were eligible to participate. Interview memos were analyzed concurrently with data collection to refine the measure in stages.

**Results:**

We interviewed 26 participants. Items were tested over seven stages and led to four main changes. First, we revised three items to reflect concepts perceived as important to the specific decision-making context of abortion. Second, we removed two items that emerged as potentially inappropriate for this context. Third, we modified language in four items to improve their appropriateness for this context (e.g., ‘telling me’ to ‘explaining’; ‘letting me say’ to ‘listening to’). Fourth, we modified language in three items to improve their clarity. Three items remained unchanged, as there was consistent agreement on their importance, understandability, and relevance.

**Conclusions:**

The resulting 10-item measure, the Interpersonal Quality in Abortion Care scale, was perceived to be highly important, understandable, and feasible to complete. Future psychometric evaluation can prepare it for use in clinical practice to ensure women feel adequately informed and supported during abortion care.

## Background

Women in the United States value receiving quality interpersonal care from abortion care providers [1–6]. Interpersonal care, including the communication and rapport between the patient and provider, is a core component of patient-centered care [7, 8] and has been associated with improved health outcomes in a range of clinical conditions [9, 10]. In the abortion context, research has shown that women prioritize a patient-provider relationship that is non-judgmental [11] and responsive to their needs and preferences [11–15], which contributes to a positive abortion experience. For example, a survey of 210 surgical abortion patients found that the courtesy and support of staff and receiving individualized information were among the most important factors to women’s satisfaction with care [16].

Despite the importance of evaluating the quality of interpersonal care for patients seeking abortion, to our knowledge, no such measure is available for this context. While there are validated measures of interpersonal care quality both for patients receiving health care in general [17–19], and for patients receiving other reproductive health care [20], it is not clear whether these are suitable for application in the abortion context. Notably, because the decision to have an abortion is uniquely stigmatized [21], women’s preferences for the type of support they receive (e.g., emotional support [22], more autonomy [23]) from abortion care providers, including administrative staff, counselors, and clinicians performing the procedure, may differ from other areas of reproductive health. Also, generic measures’ content may not be as sensitive to changes in the aspects of interpersonal care that are most germane and important to abortion patients and providers [24].

The absence of a measure that can be adopted confidently to assess quality interpersonal care during abortion services has several negative implications. First, the quality of interpersonal care that women receive from different types of abortion care providers remains unknown, which is problematic given its importance as a dimension of patient-centered care [7, 8]. Second, we lack the capacity to assess the impact of existing or new approaches to delivering abortion care on women’s experiences. For example, we lack the capacity to determine whether policies that mandate the provision of controversial information during abortion counseling in certain states [25–27] facilitate or undermine interpersonal care quality. Third, we lack the capacity to assess the adequacy of current approaches to training health professionals in the provision of interpersonal care in abortion counseling [28].

To address this gap, we sought to create a patient-reported measure of interpersonal care quality that was suitable for the abortion care context. We elected to do so by adapting the Interpersonal Quality in Family Planning (IQFP) care scale [20, 29], a valid and reliable measure of interpersonal care quality during contraception services, in collaboration with end users. The IQFP contains 11-items, some of which were adopted from other measures of interpersonal care (i.e., Consultation and Relational Empathy scale [17] and the Interpersonal Processes of Care scale [30]) in three domains: interpersonal connection, receiving adequate information, and decision support. While the development [29] and validation [20] of the IQFP were rigorous and comprehensive, we felt cognitive interviews were critical to ensure the items were interpreted as intended and considered important in the context of abortion as opposed to contraception.

## Methods

The study received approval from the Dartmouth College Committee for the Protection of Human Subjects (#00030181). We adhered to the Standards for Reporting Qualitative Research (SRQR) [31].

### Study design

We conducted cognitive interviews via telephone or face-to-face depending on the participant’s preference to maximize their comfort and convenience. We adopted best practices for using cognitive interviews in measure development [32], including presenting participants with candidate items, asking them to describe what the items mean in their own words, and soliciting suggestions for improving the clarity or answerability of the items [33].

### Participants

People were eligible to participate if they were 18 years of age or older, self-identified as having had an abortion in the past year, were comfortable speaking English and/or Spanish, and lived in the US.

### Sample size and recruitment

Our target sample size was up to 30 participants, split equally between cohorts of English- and Spanish-speakers. The sample size was considered likely to be adequate to reach data saturation based on a similar study of item formulation for measure development [34]. We used purposive sampling to maximize diversity in the age, geographic location, and health literacy of participants. Specifically, we aimed to reach participants from these diverse backgrounds by distributing patient-facing study flyers and index cards at health services that offered abortion care across the US (e.g., select Planned Parenthood clinics, independent abortion providers, an academic medical center), at women’s health resource centers, and via social media of organizations working in women’s health and abortion advocacy (e.g., 1 in 3 Campaign, Our Body Ourselves, Planned Parenthood Northern New England). We also engaged a broad range of key informants working in abortion care or advocacy and requested that they share the study invitation with colleagues and via email distribution lists of organizations working in abortion care or advocacy (e.g., Abortion Care Network, Nursing Students for Choice, New Leadership Network Initiative, state Office of Sexual Health and Youth Development, and SisterReach). The study invitation requested that interested parties contact the primary author (KD) to receive patient-facing recruitment materials to share with their patients or clients. The recruitment materials, which were developed in both English and Spanish, included flyers and index cards that described the purpose of the study, the researcher leading the study, the eligibility criteria, what was involved in participating, and a phone number to express interest. Recruitment was conducted and data were collected between September and December 2017.

### Procedure

#### Demographic questionnaire

We developed a brief demographic questionnaire, which assessed participants’ age, gender identity [35], language(s) spoken at home, race, ethnicity, educational attainment [36], health insurance status [37], health literacy [38], and geographic location.

#### List of candidate items

A list of candidate items (with alternatives where relevant) was used in each cognitive interview. The first iteration of the list contained the initial adaption of items by the researchers to reference abortion methods rather than contraceptive methods (see Table 2 in *Results*). We chose not to specify the type of abortion methods so that the items could be applicable to first trimester abortion methods (i.e., medication and surgical abortion) and second trimester abortion methods (i.e., induction termination and dilation and evacuation). In some cases, minor changes were made in the language to reflect the abortion context. For example, the original IQFP item, ‘*Telling me how to take or use my birth control method most effectively*’, was initially adapted to ‘*Telling me what I need to do for my abortion*’ because ‘take or use’ and ‘most effectively’ do not correspond logically to the aspiration procedure. Also, ‘*Telling me the risks and benefits of the birth control method I chose’* was changed to ‘*Telling me the risks and benefits of my abortion method’* to omit decision-making role because some women do not have a choice of method. Finally, the opening sentence, *‘Please rate the health care provider you saw today with respect to the following qualities:’* was changed to ‘*Please rate the health professional who talked with you about abortion today on the following qualities:*’. Because women often see multiple health professionals (i.e., administrative staff, counselors, clinicians performing the procedure) in one appointment, using ‘talked with you’ was intended to focus on the dynamic that involved the most patient-provider communication, and therefore opportunity for interpersonal care. The list evolved as items were further adapted, alternatives were generated, and items finalized.

#### Interview guide and data collection

An interview guide was developed to support the cognitive interviews. This interview guide contained prompt questions to solicit participants’ thoughts and feelings about the items (e.g., “What do you think this question is asking?”, “How do you think you would be able to answer this question, and why?”, “Is there anything you find confusing or poorly worded? If so, how could we make the question easier to answer?”, “Is this an important question?”).

We planned to conduct interviews first with the cohort of English-speakers and subsequently with the cohort of Spanish-speakers, but unforeseen challenges recruiting Spanish-speakers prevented us from interviewing this group (see *Results*). The primary author (KD) conducted all English interviews and took memos to document participants’ comments, the interviewers’ reflections, and decisions about changes to the items [39]. The interviews incorporated both the think-aloud approach and question prompts to explore participants’ views on the acceptability, understandability, and importance of the adapted items [32].

Before the interview began, the interviewer reviewed the Information Sheet with prospective participants and asked them to provide verbal informed consent to participate. Participants also received a copy of the adapted measure via email or mail and were asked to carefully read over each item. During the interview, participants were asked to share what came to their mind as they reviewed the questions in the scale. At times, the interviewer also probed for clarifying questions and asked participants to consider how they would phrase items in their own words, how difficult the items were to answer, and suggestions about how we could modify the items to enhance relevance for the abortion counseling context [32].

When data saturation occurred, we consolidated feedback and presented the new iteration of the scale to the next stage of participants. At each stage, the interviewer asked participants to compare certain changes to the items in the previous version so as to confirm the changes or identify any dissention. At the end of the interview, participants completed the brief, anonymous demographic questionnaire. They received $20 upon survey completion.

#### Analytic strategy

We analyzed interview memos concurrently with data collection to iteratively refine the measure in stages. The interviewer also met periodically with researchers who had expertise in patient-reported measure development and shared decision-making to discuss the results and proposed changes to the items. She also met periodically with the research team who had developed the IQFP and had clinical training in abortion care. The objective of these discussions was to integrate a diversity in perspectives and to ensure the adapted scale remained consistent with the construct of the IQFP.

#### Reflexivity

Given the sensitivity of this research topic, the research team was mindful of how their professional background and beliefs may have influenced data collection and interpretation. Specifically, the interviewer was an academic researcher, and, to facilitate participants being more forthright in their responses, wanted to minimize perceptions of the ‘distance’ between the researcher and research participants [40]. Therefore, the interviewer let the participants ‘set the pace’ of the conversation in hopes that they would feel more comfortable with and in control of the interview process. The research team was also cognizant that our belief in the value of patients’ preferences shaped data interpretation. Specifically, when discrepancies arose between participants’ feedback and the original items, we gave precedence to participants’ suggestions because our priority was to produce a scale that aligned with patients’ needs.

## Results

### Participant characteristics

Altogether, 26 participants diverse in age, gender identity, health insurance, educational attainment, and region of residence were interviewed (see Table 1). Participants lived in nine states that represented those who were ‘extremely hostile’ (i.e., Texas, Kentucky, Tennessee), ‘hostile’ (i.e., Pennsylvania), ‘middle-ground’ (i.e., Massachusetts, New Hampshire) and ‘supportive’ (i.e., Vermont, Connecticut, California) of abortion rights according to the Guttmacher Institute policy review [41]. The interviews lasted between 15 to 60 min.

**Table 1:** Participant characteristics (*n* = 26) in the cognitive interviews

Characteristic	n
Age	
18–24	12
25–29	6
30–34	4
35–39	4
Gender	
Female	22
Female-to-Male (FTM) or Transgender Male or Trans Man	1
Genderqueer, neither exclusively male nor female	2
Additional gender category or Other	1
Educational attainment	
High school graduate or equivalent	4
College or some college	17
More than a Bachelor’s degree	5
Ethnicity	
Hispanic or Latino	1
Not Hispanic or Latino	25
Race	
White	18
Black or African American	4
Two or more races	4
Speak language other than English at home	
Yes	3
No	23
Health insurance	
Private or employee-sponsored	12
Medicaid or temporary Medicaid coverage	9
None	4
Other	1
US region of residence	
Northeast	20
South	5
West	1
Health literacy	
Adequate	24
Limited	2

*Note.* Response options with zero participants were omitted

Participants’ feedback in the cognitive interviews led to four main changes over a series of seven stages (stage 1: *n* = 6; stage 2: *n* = 5; stage 3: *n* = 4; stage 4: *n* = 5; stage 5: *n* = 2; stage 6: *n* = 2; and stage 7: *n* = 2). Table 2 provides an overview of how the items changed and the reasons underlying these revisions.

**Table 2:**
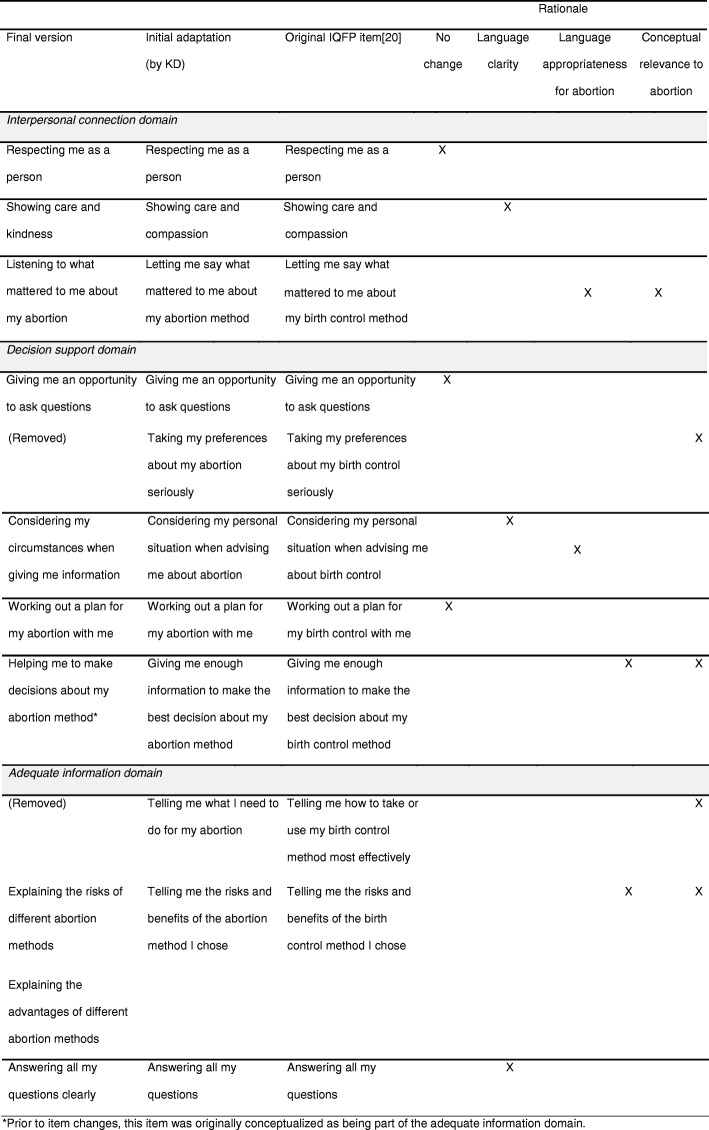
Summary of the item adaptation process and rationale for key changes

### Item adaptation outcomes

#### No changes

Three of the eleven items remained unchanged, as there was consistent agreement on their importance, understandability, and relevance. The original items were interpreted as intended. For example, ‘*Respecting me as a person’* was interpreted as providers ‘respecting my decision’, ‘not judging me’, ‘not pressuring me’, ‘taking the time to listen’*. ‘Giving me an opportunity to ask questions’* was interpreted as the patient ‘actively being invited to ask questions’ and ‘not being talked over’. *‘Working out a plan for my abortion with me’* was interpreted as the provider ‘guiding you through the process’ and ‘what you’re going to need to do and how to prepare.’

#### Conceptual relevance to the abortion decision-making context

We revised three items to reflect concepts perceived as appropriate for and important to the specific decision-making context around abortion. For example, in the initially adapted item, ‘*Letting me say what mattered to me about my abortion method*’, omitting ‘method’ was perceived to facilitate discussion of a broader range of topics, some of would relate to the method while others ranged from emotional support to scheduling. Also, the initially adapted item ‘*Giving me enough information to make the best decision about my abortion method*’ was changed to ‘*Helping me to make decisions about my abortion method’* for several reasons. First, many participants felt that the item should encompass more than ‘giving information’, which they felt was captured in the revised item ‘*Considering my circumstances when giving me information*’. There was general agreement on the importance of receiving help in making decisions and that ‘help’ was not exclusively about receiving information. Second, ‘*helping me to make decisions’* was perceived to encompass both informational and other sources of decision support, such as emotional support, which all but one woman felt was very important. We also made several substantive changes to the initially adapted item, ‘*Telling me the risks and benefits of the abortion method I chose’.* First, we separated it into two items in order to assess receipt of information about risks and benefits separately. We also replaced ‘benefits’ with ‘advantages’ because participants felt abortion methods did not confer ‘benefits’, and, instead, ‘advantages’ was perceived to be more appropriate. Participants shared that they typically had received information about the risks but not the potential advantages of a specific method, which they felt was important. As one participant shared, describing the advantages could also help combat the negativity around the decision. We also revised the items to reference receiving information about ‘different abortion methods’ instead of on just the chosen method. Participants unanimously felt it was critical for women to receive information about different methods for several reasons. First, they felt it would help women who had not yet decided on their method to become more informed and not influenced by common misconceptions. Second, they felt that for women who had already decided on their method, it would ensure that they felt fully confident in their decision. One participant felt that her providers had intentionally omitted information about one of the methods because they knew she had already decided on the alternative and thus did not want to be seen as being persuasive (#9). Lastly, participants felt that comprehensive information was important because some women decide to switch from their planned method to the alternative after counseling.

We also removed two items perceived as inappropriate for the decision-making context around abortion. We removed the initially adapted item, ‘*Telling me what I need to do for my abortion’,* because it was perceived to be too directive in this context. Also, participants generally felt it was redundant to ‘*Working out a plan for my abortion with me*’, which they preferred. As one participant explained, ‘*Telling me what I need to do for my abortion’* seemed only about what the patient needs to do, whereas ‘*Working out a plan for my abortion with me*’ felt more like a balanced exchange about both the patient’s and provider’s responsibilities (#3). We also removed the initially adapted item, ‘*Taking my preferences about my abortion seriously’*, because there was consistent confusion about what ‘preferences’ referred to in the abortion decision-making process. Participants often assumed it meant preferences for whether to have an abortion instead of about the abortion method. Some participants also shared that they knew nothing about abortion before speaking with the abortion counselor, so they did not have any preferences and therefore felt that they would have had a hard time answering this item. In addition, this item became redundant to the initially adapted item ‘*Listening to what mattered to me about my abortion*’, which was perceived by several participants to encompass engagement with preferences.

#### Language appropriateness for the abortion decision-making context

We modified the language in four items to improve their appropriateness for the abortion context, which mostly stemmed from perceptions that the language was too directive when related to abortion. For example, we revised the original item, *‘Considering my personal situation when advising me about birth control’,* to ‘*Considering my circumstances when giving me information*’ based on feedback that ‘advising’ was too directive in this context. One participant described that ‘advising me’ felt like she was going to see someone who would tell her what she should do, instead of someone who could help her to make sense of what was going on and to make the right decisions for her (#20). Also, following the initial adaptation to the item, ‘*Telling me the risks and benefits of the abortion method I chose’, ‘*telling me’ was changed to ‘explaining’ because some participants felt ‘telling me’ was too ‘aggressive’. Similarly, following the initial adaptation of the item, ‘*Letting me say what mattered to me about my abortion method*’, ‘letting me say’ was changed to ‘listening to’ in order to achieve a more supportive connotation. For example, one participant explained that she preferred ‘listening to*’* because she felt it implied being heard and respected whereas ‘letting me say’ did not imply ‘active listening’ (#6). In the item, ‘*Giving me enough information to make the best decision about my abortion method*’, participants felt that qualifying the decision as the ‘best decision’ was not appropriate.

#### Language clarity for general understandability

We modified the language in three items to improve their clarity and users’ general understanding of the concepts. For the original item *‘Showing care and compassion’*, some participants did not understand the meaning of ‘compassion’, and others felt ‘compassion’ and ‘care’ were too similar. Therefore, the consensus was that ‘kindness’ was more straightforward and better complemented ‘care’. Due to consistent confusion around the meaning of ‘personal situation’ in the initially adapted item, *‘Considering my personal situation when advising me about abortion’*, we opted to use ‘circumstances’, which was perceived to be more understandable and inclusive of any topic that a woman may want to discuss. For the original item, ‘*Answering all my questions’*, participants felt that adding ‘clearly’ was important to ensure the answers were provided in a way that the patient understands (e.g., ‘slowly and completely’).

#### Scale acceptability

Participants unanimously agreed on the importance and relevance of measuring interpersonal care quality during abortion care. While items pertaining to interpersonal connection and information provision were consistently perceived to be important and relevant, there were a few outliers pertaining to decision support. For example, after reflecting on the revised item, ‘*Helping me to make decisions about my abortion’*, one participant expressed a desire for providers to only give her information because she did not want help to make her decision, and instead wanted them to be neutral (#22). When probed about whether this verbiage undercut women’s agency in the decision-making process, other participants felt it was appropriate and important because, as one participant shared, there will always be some medical decisions that benefit from providers’ input (#26). In addition, after considering the revised item, ‘*Listening to what mattered to me about my abortion’*, one participant explained that she did not want counseling for emotional support so was not looking for her providers to listen about what mattered to her. However, she felt the question still applied to her and would have given them the highest possible score because her providers respected her boundaries by not inquiring about her feelings (#12).

Participants often shared that they had not expected to receive aspects of care described in some of the items. For example, when one participant discussed her reaction to the original item, ‘*Respecting me as a person*’, she explained that she had not thought about this before, but felt it was a very important question for women to consider (#15). Some participants wished that they had access to these questions during their abortion experience to shape their expectations of care. For example, when asked about the initially adapted item, ‘*Working out a plan for my abortion with me’*, one participant explained how much she liked this question and wished she had known developing a plan with her providers was a possibility. She shared that, at the time of her abortion, she felt that she did not deserve that kind of support and therefore was not expecting it, so she may not have understood how to answer this question (#20).

#### Scale completeness

When asked if there was anything missing, the few suggestions included an item about legal requirements for counseling and an item about trustworthy after care resources.

#### Spanish translation

Due to unforeseen challenges with recruitment, we were unable to conduct cognitive interviews with Spanish speakers. Therefore, we opted for translation of the IQAC scale into Spanish by bilingual researchers with expertise in Spanish translation, abortion counseling, and patient-reported measure development, including the Spanish version of the IQFP. The IQAC scale was first independently translated to Spanish by one researcher (RR) and then back translated by a second researcher. Revisions were made to the translated scale based on discussion between the researchers until agreement was achieved. The final scale was reviewed for comprehension with a third native Spanish-speaking researcher (DA).

## Discussion

This qualitative study developed a new patient-reported measure, the Interpersonal Quality in Abortion Care (IQAC) scale, which was perceived by participants to be highly important, understandable, and feasible to complete. The results have several implications. First, the IQAC scale is unprecedented in the abortion field, and has potential to be used in routine care to ensure providers adequately inform and support women in their abortion counseling experience. While women often report counseling as helpful, positive experiences are not universal [42], including in our study, where some participants raised concerns that they did not receive adequate informational support. Therefore, receiving patient-reported data may be an effective strategy for motivating providers to change their approach to address gaps in their performance and improve interpersonal care [43, 44].

In addition to these effects on provider behavior, our results also suggest that the IQAC scale could be a useful tool for shaping patients’ expectations and behavior. Specifically, the process of completing patient-reported outcome measures can facilitate patients to develop expectations for a positive care experience and support information sharing and discussion with their provider [45]. This is particularly relevant to the abortion context, because women face barriers expressing their counseling preferences [23] due to the stigma surrounding this experience [46]. Indeed, in our study, some participants did not expect to be treated with respect or to be able to create a comprehensive plan for their abortion with their provider. Thus, completing the IQAC scale at the end of counseling could serve as a tool to empower women to better understand what aspects of interpersonal care are possible and express any outstanding concerns or questions. With more active patient participation, providers may better understand patients’ definitions of quality interpersonal care and align their counseling approach accordingly. In turn, patients may receive more tailored counseling (e.g., emotional support, which some patients value [22] while others reject [23]) and feel more informed, both important factors to women’s satisfaction with their abortion experience [47].

This study also has implications on the development of patient-reported outcomes measures more broadly. Specifically, it highlights the tension that can arise when patients’ preferences are discordant with previously tested and validated measures. Ultimately, by prioritizing patient perspectives, items in the final measure diverged from items in the IQFP and Consultation and Relational Empathy scales [17] in both wording and the underlying domains. For instance, participant feedback led to the substantive revision of an item to encompass both informational and other sources of decision support, such as emotional support (i.e., ‘*Giving me enough information to make the best decision about my abortion method*’ to ‘*Helping me to make decisions about my abortion method’*). Also, verbiage seen as patient-centered and appropriate during contraceptive care (i.e., ‘advising me’ and ‘telling me’) was changed based on feedback that it felt more problematic in the abortion context. Although some researchers believe modifying measures for a different population threatens their reliability and validity [48], others believe that if a measure is not suitable in the new context (e.g., meaning of the concept or items differ, not interpreted as intended), it will produce erroneous results [49]. Because of the important sociopolitical contextual factors surrounding abortion, we felt prioritizing participants’ feedback was justified and provides indirect evidence of the IQAC scale’s face validity [50]. We also acknowledge, however, that it remains unclear how these changes affect the scale’s psychometric properties. Therefore, a psychometric evaluation of the IQAC scale is an important next step in producing a valid and reliable tool for assessing the interpersonal care quality of abortion care.

### Limitations and strengths

This study has several limitations and strengths. First, recruiting a convenience sample may have introduced selection bias, possibly leading people to choose to participate who have had more positive or negative abortion experiences than the typical patient. However, these more extreme experiences likely make women more sensitive to the meaning and wording of the items, and therefore allowed us to integrate perspectives of those who would be more discerning when answering the scale in the real-world. Second, the few dissenting views about the importance of certain items related to decision support suggests that this aspect of the measure may not be relevant to all patients, and thus raises questions about the generalizability of the scale’s acceptability. While our results indicate that women who are not looking for decision support may still be able to answer these items in a meaningful way, the use of a ‘not applicable’ option may be worth considering as an area of further investigation. Third, we did not assess in which trimester participants had had their abortion, and thus were unable to explore variation in acceptability by this characteristic, so we also suggest this topic as an area of further investigation. Finally, in a deviation from our plans, the Spanish translation of the scale did not undergo cognitive interview testing, so it is critical that further research be conducted with Spanish-speakers to explore its acceptability for this subpopulation. Our sample was also predominantly white with higher educational attainment, therefore exploring the scale’s acceptability with underrepresented populations is also recommended. The strengths of this study include using a sampling methodology and interview techniques (e.g., choice of setting) to facilitate participation from a population that traditionally has been hard to engage in research. Also, the decision to adapt the IQFP for the abortion context using cognitive interviews is both rigorous and more practical than creating a measure de novo [49], and provides the opportunity to more consistently compare interpersonal care quality in contraceptive and abortion care. Finally, this study provides novel insights into what aspects of interpersonal care quality women desire, which can inform abortion counseling practices and quality improvement initiatives.

## Conclusions

In collaboration with end-users, this study produced the IQAC scale, a new patient-reported measure of the quality of interpersonal care in abortion services. This 10-item measure was perceived by participants to be highly important, understandable, and feasible to complete. Such a measure is unprecedented in the abortion field, and has potential to serve as a tool to ensure women feel adequately informed and supported in their abortion care experience. Therefore, a psychometric evaluation of the IQAC scale is an important next step in producing a valid and reliable tool for assessing the interpersonal quality of abortion care.

## Abbreviations

IQAC: Interpersonal Quality in Abortion Care
IQFP: Interpersonal Quality in Family Planning
